# Experimental infection and transmission of *Leishmania* by *Lutzomyia cruzi* (Diptera: Psychodidae): Aspects of the ecology of parasite-vector interactions

**DOI:** 10.1371/journal.pntd.0005401

**Published:** 2017-02-24

**Authors:** Everton Falcão de Oliveira, Elisa Teruya Oshiro, Wagner de Souza Fernandes, Paula Guerra Murat, Márcio José de Medeiros, Alda Izabel Souza, Alessandra Gutierrez de Oliveira, Eunice Aparecida Bianchi Galati

**Affiliations:** 1 Programa de Pós-Graduação em Saúde Pública, Faculdade de Saúde Pública, Universidade de São Paulo, São Paulo, São Paulo, Brasil; 2 Departamento de Estatística, Campus Macaé, Universidade Federal do Rio de Janeiro, Macaé, Rio de Janeiro, Brasil; 3 Centro de Ciências Biológicas e da Saúde, Universidade Federal de Mato Grosso do Sul, Campo Grande, Mato Grosso do Sul, Brasil; 4 Faculdade de Medicina Veterinária e Zootecnia, Universidade Federal de Mato Grosso do Sul, Campo Grande, Mato Grosso do Sul, Brasil; 5 Departamento de Epidemiologia, Faculdade de Saúde Pública, Universidade de São Paulo, São Paulo, São Paulo, Brasil; Fundaçao Oswaldo Cruz, BRAZIL

## Abstract

Several parameters should be addressed before incriminating a vector for *Leishmania* transmission. Those may include its ability to become infected by the same *Leishmania* species found in humans, the degree of attractiveness for reservoirs and humans and capacity to sustain parasite infection under laboratory conditions. This study evaluated the vectorial capacity of *Lutzomyia cruzi* for *Leishmania infantum* and gathered information on its ability to harbor *L*. *amazonensis*. Laboratory-reared *Lu*. *cruzi* were infected experimentally by feeding them on dogs infected naturally with *L*. *infantum* and hamsters infected with *L*. *amazonensis*. Sand fly attractiveness to dogs and humans was determined using wild caught insects. The expected daily survival of infected *Lu*. *cruzi*, the duration of the gonotrophic cycle, and the extrinsic incubation period were also investigated for both parasites. Vector competence was investigated for both *Leishmania* species. The mean proportion of female sand flies that fed on hosts was 0.40. For *L*. *infantum* and *L*. *amazonensis*, *Lu*. *cruzi* had experimental infection rates of 10.55% and 41.56%, respectively. The extrinsic incubation period was 3 days for both *Leishmania* species, regardless of the host. Survival expectancy of females infected with *L*. *infantum* and *L*. *amazonensis* after completing the gonotrophic cycle was 1.32 and 0.43, respectively. There was no association between *L*. *infantum* infection and sand fly longevity, but *L*. *amazonensis*–infected flies had significantly greater survival probabilities. Furthermore, egg-laying was significantly detrimental to survival. *Lu*. *cruzi* was found to be highly attracted to both dogs and humans. After a bloodmeal on experimentally infected hosts, both parasites were able to survive and develop late-stage infections in *Lu*. *cruzi*. However, transmission was demonstrated only for *L*. *amazonensis*–infected sand flies. In conclusion, *Lu*. *cruzi* fulfilled several of the requirements of vectorial capacity for *L*. *infantum* transmission. Moreover, it was also permissive to *L*. *amazonensis*.

## Author summary

Leishmaniasis is a vector-borne disease caused by the *Leishmania* parasite. It is transmitted by phlebotomine sand flies when infected females take a bloodmeal from a mammalian host. The transmission of *Leishmania* species involves complex ecological interactions between parasite–vector and vector–host. Until recently, the sand fly *Lutzomyia longipalpis*, which belongs to a species complex, had been solely incriminated in Latin American transmission of *Leishmania infantum*. However, there are still uncertainties concerning the *L*. *infantum* transmission, especially in an urban area endemic for visceral leishmaniasis in Brazil. Along the years, *Lutzomyia cruzi* has been suspected of transmitting *L*. *infantum* in Corumbá and vicinity, and this study aimed to provide more accurate evidence of this phenomenon. Laboratory-reared *Lu*. *cruzi* were infected experimentally by allowing them to feed on dogs naturally infected with *L*. *infantum* and on hamsters infected with *L*. *amazonensis*. Our results identified important life cycle parameters of *Lu*. *cruzi* infected by *Leishmania* in laboratory conditions. We identified the components related to vectorial capacity, and also the vector incrimination criteria of *Lu*. *cruzi* for *L*. *infantum*. Additionally, we demonstrated the experimental transmission of *L*. *amazonensis* by *Lu*. *cruzi* to a naïve host, and revealed strong evidence that supports *Lu*. *cruzi* as a vector of *L*. *infantum* and as a permissive vector for *L*. *amazonensis*.

## Introduction

The parasite *Leishmania infantum*, the causative agent of visceral leishmaniasis (VL), is commonly transmitted by *Lutzomyia longipalpis*, which is widely distributed in Latin America [1,2]. In Brazil, it is responsible for most of the VL cases throughout the country [2]. However, VL cases due another vector have been reported in regions where *Lu*. *longipalpis* is absent [3–8]. In Corumbá (State of Mato Grosso do Sul, Brazil) and surrounding areas, *Lutzomyia cruzi* has been incriminated as a suspected vector of *L*. *infantum* on the basis of eco-epidemiological evidence supported by the high frequency of parasite transmission in urban areas, anthropophily, overlap of the temporal and spatial distributions of the vector and the human and canine cases of VL [3], and findings of sand flies naturally infected by *L*. *infantum* [4,9].

The designation of a sand fly species as a vector of *Leishmania* must meet the criteria initially proposed by Killick-Kendrick [10], namely: (i) the existence of a substantial relationship with reservoirs and humans (demonstrating that the sand fly is anthropophilic and commonly feeds on the reservoir host(s)); (ii) the repeated isolation and identification, from female sand flies that have not recently fed, of the same species of *Leishmania* as is found in human cases of leishmaniasis; (iii) the presence of the sand fly in places where *Leishmania* and the disease it causes are found; (iv) the density and rate of natural infection by *Leishmania*; (v) the development of late-stage infections in specimens experimentally infected in the laboratory; and (vi) the transmission of the parasite to a susceptible host during a bloodmeal. Moreover, mathematical modeling has recently been employed to demonstrate how vector abundance influences the incidence of the disease [11].

The vectorial capacity of a hematophagous insect is defined as the expected number of potentially infectious bites that would occur from the total number of insects that bite a single perfectly infectious source (or host) on a single day, assuming all the females that took a bloodmeal become infected [12,13]. The first mathematical models for estimating vectorial capacity were proposed by Macdonald [14] and Garret-Jones [12] for *Anopheles* spp. These models considered vector density in relation to the host, attraction to the host, the proportion of female insects that fed on the host, the estimated probability of survival in the field, the duration of the gonotrophic cycle and the extrinsic incubation period, and the proportion of infected females with infective forms of the parasite. Although some of these parameters are important in fulfilling the Killick-Kendrick et al. criteria [10] since they relate to the biological criteria of vector incrimination, until now there are few published reports focused on the vectorial capacity of sand flies [15,16]. Furthermore, some biological aspects of *Leishmania*–infected *Lu*. *cruzi*, including gonotrophic cycles, mortality, and survival, are still unknown, especially when they have fed on different blood sources.

Except when a vector is confirmed, frequent findings of sand flies naturally infected by other species of *Leishmania*, along with the ability of flies to support the development of these other species, have led researchers to categorize them as permissive vectors [17], as occurs with *Lu*. *longiapalpis*, which is experimentally permissive for various *Leishmania* species, including *L*. *amazonensis* [18], *L*. *mexicana* [19], and *L*. *peruviania* [20]. The existence of permissive vectors demonstrates the need to investigate other species with the potential to transmit *Leishmania*, especially in areas that have cases of VL when the confirmed vector is absent.

Based on previous eco-epidemiological observations [3,4,9], we investigated the current status of *Lu*. *cruzi* as a possible vector of *L*. *infantum* in Corumbá, Brazil, as well as some of its biological parameters in relation to vectorial capacity. The ability of *Lu*. *cruzi* to sustain infection by *L*. *amazonensis* was also assessed as a means of understanding its permissiveness to different *Leishmania* species.

## Methods

### Establishment of the sand fly colony

To establish a colony and acquire first generation (F_1_) adults, wild specimens of *Lu*. *cruzi* were collected from the urban perimeter of the municipality of Corumbá (19° 00′ 33″ S; 57° 39′ 12″ W; 118 m above sea level), which is located in the northeastern part of the State of Mato Grosso do Sul (central-western Brazil), in the Pantanal wetland region on the border of Bolivia.

Sand flies were collected manually with the aid of electric aspirators [21] in and around chicken coops located in the peridomicile of a private residence (19° 0′ 45.49″ S; 57° 37′ 30.90″ W), and with CDC light traps deployed between 18:00 and 06:00. To ensure survival after collection, the insects were placed in nylon cages with metal frames (30 × 30 × 30 cm) that were enveloped in moistened towels to maintain humidity. Apple slices were placed in each cage to serve as a food source until the flies could be provided with a bloodmeal from hamsters (*Mesocricetus auratus*). The specimens were identified according to the classification system of Galati [22].

The colony was established and maintained at the Human Parasitology Laboratory of the Federal University of Mato Grosso do Sul (Brazil) according to the protocol described by Oliveira et al. [23].

### Experimental infection of *Lutzomyia cruzi* by *Leishmania infantum* and *L*. *amazonensis*

The experimental infection trials of *Lu*. *cruzi* were made by xenodiagnoses performed on dogs and hamsters. For experimental infection by *L*. *infantum*, six naturally infected dogs were obtained from the Zoonosis Control Center in the municipality of Campo Grande. All animals had specific symptoms of canine visceral leishmaniasis, in addition to having positive serological and direct parasitological tests for *Leishmania*.

The experiment with dogs was repeated six times. Each experiment was performed with a different animal and always between 18:00 and 19:00. The dog was sedated with an intramuscular injection of 40 to 80 mg/kg sodium thiopental and then placed in a nylon cage (1.90 × 0.90 × 1.80 m) divided into two compartments (S1 Fig). First generation (F_1_) *Lu*. *cruzi* males and females aged 3–4 days were then released into the cage. After 60 min, all the flies were aspirated with a hand-held electric insect collector and transferred to a smaller nylon cage (30 × 30 × 30 cm) that was wrapped in a moistened towel. After 24 h, each female was removed from this cage and placed in an acrylic jar (2.5 cm diameter × 3.5 cm height) floored with plaster. The jar was closed with a nylon cloth under a plastic lid with a hole in the center (1.20 cm in diameter). Small cubes of apple were placed on the nylon cloth in the central hole as a source of food. The jars were kept in a rectangular polyethylene recipient (34 × 14 × 10 cm) lined on the bottom with moistened filter paper and kept with the lip partially open in a B.O.D. incubator (MA-414, Marconi, São Paulo, SP, BRA) at a temperature of 27 to 28°C and a relative humidity higher than 80%. Only some of the females were given a bloodmeal on a host, thereby allowing us to form two groups of females, one with blood in their guts (engorged) and the other without blood in their guts (non-engorged). The jars of both groups were inspected three times a day (08:00, 14:00, and 20:00) to determine mortality and egg-laying capacity, as well as daily survival rates (life expectancy after xenodiagnosis) and the status of the gonotrophic cycle (median number of days between the bloodmeal and egg-laying).

For infection by *L*. *amazonensis*, two hamsters were experimentally infected with strain IFLA/BR/1967/PH8 through an intradermal injection in the plantar pad of the back paws. The parasite was provided by the Leishmaniasis Laboratory of the René Rachou Research Center (CPqRR/Fiocruz-BH). When the xenodiagnoses were performed, the animals exhibited nodules on the back paws and snout.

The same method used for the xenodiagnosis of dogs was used for the experimental infection of *Lu*. *cruzi* by *L*. *amazonensis*. The only difference was the size of the cage (30 × 30 × 30 cm) used for the exposure of the animals to the sand flies. Three replicates were performed. The second and the third replicates were performed using the same animal.

### Infection rate and extrinsic incubation period

The infection rate was determined by dissection of the females that died each day as well as by molecular analysis. The guts of these females were examined under optical microscopy at a magnification of 400×. *Leishmania* DNA was tested for using a polymerase chain reaction (PCR). In the absence of naturally dead females, two or three engorged females were sedated and dissected to evaluate the extrinsic incubation period in relation to the parasite.

The dissection method described by Johnson et al. [24] was used to expose the gut and spermathecae for subsequent study of the presence of flagellates and confirmation of the sand fly species, respectively. After dissection and inspection for flagellates, the contents of the slide and the gut of specimens both with and without flagellates were separately transferred to 1.5-mL microtubes containing isopropyl alcohol. On some occasions, especially when there was heavy infection by flagellates, after the material was transferred to the microtube, we attempted to fix and stain the residual content on the slide with Errecart solution and Giemsa stain, respectively.

The infection rate of *Lu*. *cruzi* by each species of *Leishmania* was calculated as the ratio of the number of females with positive PCR results to the total number of engorged females. The frequency of potentially infective females was determined from the ratio of the number of females presenting infective forms (metacyclic promastigote) to the total number of females infected with any promastigote morphotypes in the foregut (head), stomodeal valve, and/or anterior thoracic midgut [15]. Metacyclic promastigotes were distinguished by morphology (elongated body with flagellar length ≥3-times body length), location (foregut and anterior midgut), and motility [19,25,26]. Parasite loads were graded according to Myskova et al. [27].

### Molecular detection and identification of *Leishmania* infection in female sand flies

To confirm infection by *Leishmania*, all females used in the experimental infection by xenodiagnosis were screened by PCR. The contents of the slide and the digestive tube were ground using a plastic pestle in 1.5-mL tubes with 300 μL of 5% *Chelex* resin solution (Bio-Rad, Hercules, CA, USA). DNA extraction was performed according to the protocol described by Oliveira [28].

PCR was performed targeting an approximately 300-base-pair (bp) region of the internal transcribed spacer of the *Leishmania* ribosomal gene (ITS1), as previously described by El Tai et al. [29] and Oliveira et al. [28]. The positive controls were DNA from *L*. *infantum* (MHOM/BR/1972/BH46) and *L*. *amazonensis* (IFLA/BR/1967/PH8) extracted from cultures. The PCR products from positive samples were subjected to *Hae*III restriction enzyme digestion, according to Schönian et al. [30]. One μL of 10× buffer, 1 unit of *Hae*III enzyme, and 1 μg of DNA from the PCR analysis were used, and the volume was completed with 10 μL of ultrapure water. The sample was incubated in a water bath at 37°C overnight. The material was then submitted to electrophoresis on a 2% agarose gel with TBE buffer for 3 hours.

### Survival

The nonparametric Kaplan-Meier estimator was used to estimate the survival rates of: (1) females that had fed on blood (engorged); (2) females that had not fed on blood (non-engorged); (3) infected females; and (4) non-infected females. Mean and median values were calculated from the estimated curves. Survival curves were compared using the log-rank test. According to Colosimo and Giolo [31], the nonparametric Kaplan-Meier estimator is defined by:

$$\hat{S}\left(t\right)=\prod_{j:t_{j}<t}\left(\frac{n_{j}-d_{j}}{n_{j}}\right)=\prod_{j:t_{j}<t}\left(1-\frac{d_{j}}{n_{j}}\right),$$

where:

- *t*_1_ < *t*_2_ < ⋯ < *t*_*k*_ are *k* distinct times ordered by the time that failure (death of a female) occurred;
- *d*_*j*_ is the number of failures at *t*_*j*_, *j* = 1, 2, …, *k*; and
- *n*_*j*_ is the number of living females (at risk) at *t*_*j*_, *j* = 1, 2, …, *k* (females that had not died or had death induced up to the day prior to *t*_*j*_).

With respect to the number of missing observations (females anesthetized for dissection), the most adequate estimate of the median was determined by linear interpolation of the Kaplan-Meier curve. Mean survival time was estimated by:

$$\hat{t_{m}}=t_{1}+\sum_{j=1}^{k-1}\hat{S}\left(t_{j}\right)\left(t_{j+1}-t_{j}\right),$$

where:

- *t*_1_ < *t*_2_ < ⋯ < *t*_*k*_ are *k* distinct times ordered by the time that failure (death of a female) occurred; and
- $\hat{S}\left(t\right)$ is the Kaplan-Meier estimator [31].

In addition, the Cox regression model was used to study the relationship of survival rates to the covariates of *Leishmania* infection, egg-laying, egg-laying time after the bloodmeal (gonotrophic cycle), and number of eggs per female that performed egg-laying.

The general expression of the Cox regression model considers equality as:

$$\lambda \left(t\right)=\lambda_{0}\left(t\right)g\left(x\prime \beta \right),$$

where:

- λ(*t*) is the failure rate at time *t*; and
- *g* is a non-negative function that must be specified, such that *g*(0) = 1.

The form of *g*(**x**′***β***) adopted in this analysis was *g*(x′*β*) = exp(**x**′***β***) = exp(*β*_1_*x*_1_ + *β*_2_*x*_2_ + ⋯ + *β*_*p*_*x*_*p*_), with the ***β*** parameters of the vector being associated with the explanatory variables (covariates).

The analysis was conducted using R software version 3.3.0 [32] and by employing a 5% (α = 0.05) significance level.

### Human and canine attractiveness to sand flies

Human and dog attractiveness to sand flies was evaluated by monthly experiments conducted between March 2013 and February 2014. The method described by Pinto et al. [33] was employed, which consists of using impermeable polyester tents (205 × 145 × 105 cm) with two screened orifices to allow for the circulation of air: one at the entrance and one at the exit (S2 Fig). At the orifice for the entrance of air, a fan was coupled to a device to control the velocity of air introduced to the tent. Outside the tent in front of the orifice for exiting air, an automatic CDC trap without a light was deployed to capture sand flies attracted to the expelled air.

Two tents deployed from 18:00 to 06:00 were used in each experiment. A human (the researcher and first author, EFO) remained in one tent and a dog was placed in the other. Both tents were set up at the same time in the same peridomicile area at a distance of approximately 18 m from one another over two consecutive nights. The position of the tents was altered on the second night. Forty-eight replicates were performed to evaluate human and canine attractiveness to the target species. Only one dog (not infected by *L*. *infantum*) was used in this experiment, the participation of which was authorized by its owner.

As an alternative to the initial method, canine attractiveness was also evaluated by collection (manual aspiration) directly from dogs. To minimize the possible interference of human attractiveness, a 5-min collection cycle on dogs and the surrounding area (kennel) was performed, followed by an interval of 10 min. Moreover, an attempt was made to adapt the collection method using a Disney trap [34], with the aid of metallic discs (35 cm in diameter) greased with castor oil on the surface. Three discs were attached with double-sided adhesive tape to the inner walls of three kennels for three nights. Both methods (aspiration and collection on metallic discs) were performed in the peridomiciliary area of three private residences in urban areas in the municipality of Corumbá. The owners of these residences gave permission for the study to be conducted at these sites.

### Experimental transmission of *Leishmania* spp.

To demonstrate the vector competence of *Lu*. *cruzi* through the experimental transmission of *Leishmania* spp., we used the F_1_ females that were used in xenodiagnosis experiments.

After becoming engorged on infected hosts (1 day after the extrinsic incubation period), the surviving females on day 4 were challenged to bite naïve hamsters in nine experiments. Table 1 shows the number of females challenged to bite both infected (first bloodmeal) and naive hosts (second bloodmeal), as well as the number of engorged females in each blood feeding, grouped according to parasite. Male and female hamsters aged 30 to 40 days were used. All attempts at experimental transmission of the parasite were performed between 18:00 and 19:00 in nylon cages with metal frames (30 × 30 × 30 cm) covered with dark cloth.

**Table 1:** Number of females exposed (1) in xenodiagnoses, and (2) to susceptible hosts, by species of *Leishmania* and infected host.

Animal / species of *Leishmania*	Females exposed in xenodiagnosis	Females that fed during xenodiagnosis (first bloodmeal)	Females exposed to susceptible host	Females that fed on susceptible host (second bloodmeal)	Infected females^a^
**Dog / *L*. *infantum***					
1	16	12	7	1	0
2	11	7	6	0	0
3	5	4	4	0	0
4	7	7	5	2	0
5	143	76	37	26	13
6	458	150	85	42	0
**Hamster / *L*. *amazonensis***					
1	95	37	21	6	3
2	41	16	2	2	1
3	56	24	6	6	4

^a^Infected females among those that made the second bloodmeal.

#### Maintenance and examination of hamsters

Whether infected or not, all hamsters exposed to insect bites were maintained in cages with sterilized sawdust on a ventilated rack equipped with mini-isolators, and they were given free access to food and water. The cages were cleaned weekly, and the hamsters were examined for their general physical state (weight loss, skin integrity, and changes in fur state). Six months was the maximum follow-up period for the evaluation of possible *Leishmania* infection. When clinical signs suggestive of infection were found, the affected hamster was euthanized using carbon dioxide, and a necropsy was performed to remove the spleen. Spleen tissue samples were used to prepare imprint slides with the Giemsa staining method (direct diagnosis) as well as for seeding in a culture medium, and were then stored in microtubes at -20°C for further identification of *Leishmania* DNA using PCR.

#### Isolation of parasites and molecular detection of *Leishmania* infection in hamsters

To isolate the parasites, the samples obtained during necropsy were seeded in artificial Neal-Novy-Nicolle medium with the liquid phase of Schneider’s Insect Medium (Sigma-Aldrich, St Louis, MO, USA) supplemented with 20% fetal bovine serum (Cultilab, Campinas, SP, BRA) and 140 μg/mL gentamicin (Sigma-Aldrich), and maintained in an incubation chamber at 25°C. After the seventh day, the cultures were examined weekly under light microscopy for four consecutive weeks.

PCR analysis was performed on the samples and cultures (when positive). The parasite was identified using restriction fragment length polymorphism analysis (PCR-RFLP). DNA was extracted from the samples with the Wizard DNA Purification Kit (Promega, Madison, WI, USA), following the manufacturer’s instructions. We used the same PCR and PCR-RFLP method described for the analysis of engorged females.

The sequencing of PCR products amplified and identified as *L*. *amazonensis* by PCR-RFLP was carried out in both directions using the ABI Prism BigDye Terminator v3.1 Cycle Sequencing Kit (Applied Biosystems, Foster City, CA, USA). The protocol of the sequencing reaction was previously described by Oliveira et al. [35]. Sequencing was only performed on samples positive for DNA of *L*. *amazonensis*, as this was the first description of experimental transmission of this parasite by *Lu*. *cruzi*.

### Ethical statement

This study received approval from the Animal Experimentation Ethics Committee of the Federal University of Mato Grosso do Sul (Brazil) under process number 491/2013. The research group has a permanent license for the collection of zoological material issued by the Brazilian Institute of the Environment and Renewable Natural Resources (IBAMA: SISBio 25952–1). The field studies were carried out on private land, and the owner gave permission to conduct the study in his peridomicile area. In addition, the field studies did not involve endangered or protected species.

The dogs used for the experimental *Lu*. *cruzi* infection were naturally infected by *L*. *infantum*. These animals were voluntarily surrendered by their owners to the Zoonosis Control Center for euthanasia (the recommended procedure by the Ministry of Health of Brazil [36]); the Animal Experimentation Ethics Committee of the Federal University of Mato Grosso do Sul approved the use of these animals. Therefore, besides the approval of the Ethics Committee, the inclusion of the animals in the study was conditional on the authorization signed by the owner of the animal and by the Zoonoses Control Center (the institution responsible for the animal after the voluntary surrender). After xenodiagnosis, the dogs were returned to the Zoonosis Control Center. This procedure was carried out by this institution as a routine service and on the recommendation of the Visceral Leishmaniasis Control Program of the Ministry of Health of Brazil [36].

The Zoonosis Control Center was responsible for housing conditions prior to and after xenodiagnoses. The center ensured access to food and water, environmental enrichment, biosafety considerations for infected animals, and made all efforts to alleviate suffering.

The human included in the study to assess attractiveness to sand flies was the study’s first author (EFO), and there was no inclusion of other humans. Thus, approval of the study by an Ethical Review Board was not necessary, since the possible risks of this experiment were equivalent to the risks inherent in field studies. In addition, it is important to highlight that the tents used were completely sealed, and there was no exposure or contact between the host (human or dog) and the sand flies during the experiment.

## Results

### Experimental infection, gonotrophic cycle, and extrinsic incubation period

A total of 832 *Lu*. *cruzi* females were exposed to infected hosts, and 333 of them fed during the experiments (Table 2). In the experiments with dogs, the proportion of female sand flies that fed ranged from 0.33 to 1.00, with a total value of 0.40 and a mean of 0.68. This proportion varied directly in relation to the number of species used in each replicate, and considerably diminished in the last two replicates. The feeding proportion in the experiments with infected hamsters ranged from 0.39 to 0.43, with a total and a mean of 0.40.

**Table 2:** *Lutzomyia cruzi* females that fed during xenodiagnoses, according to host and species of *Leishmania*.

Experiments	Dogs infected with *L*. *infantum*			Hamsters infected with *L*. *amazonensis*		
	Exposed females	Engorged females	Proportion of engorged females	Exposed females	Engorged females	Proportion of engorged females
1	16	12	0.75	95	37	0.39
2	11	7	0.64	41	16	0.39
3	5	4	0.80	56	24	0.43
4	7	7	1.00	…	…	…
5	143	76	0.53	…	…	…
6	458	150	0.33	…	…	…
**Total**	640	256	0.40	192	77	0.40

… experiment not performed.

Considering all the engorged female sand flies, regardless of bloodmeal source, the median length of the gonotrophic cycle was 6 days. For females that fed on dogs, the median length of the cycle was also 6 days (mean = 6.52; SD = 2.76; minimum = 3; maximum = 15 days). However, for the females that fed on hamsters, the cycle was 5 days (mean = 5.73; SD = 1.62; minimum = 4; maximum = 9 days).

Table 3 displays the number of engorged, dissected, infected, and potentially infective female sand flies by host and species of *Leishmania*.

**Table 3:** Number of engorged, dissected, infected, and potentially infective *Lutzomyia cruzi* females in relation to host, number of days after the xenodiagnosis, and species of *Leishmania*.

Days after xenodiagnosis	Dogs infected with *L*. *infantum*			Hamsters infected with *L*. *amazonensis*		
	Dissected	Infected by microscopic exam (pot. inf.)	Infected by PCR	Dissected	Infected by microscopic exam (pot. inf.)	Infected by PCR
1	31	0 (0)	0	6	0 (0)	0
2	15	0 (0)	0	13	0 (0)	1
3	19	2^a^ (2^a^)	2	10	5^b^^,^^c^ (5^b^^,^^c^)	5
4	19	6 (6)	6	11	5 (5)	6
5	38	2 (2)	5	15	7 (5)	9
6	23	1 (1)	3	8	2 (1)	5
7	31	3 (3)	5	7	2 (2)	3
8	32	3 (3)	4	4	1 (1)	1
9	6	0 (0)	0	2	1 (1)	1
10	12	2 (2)	2	0	0 (0)	0
11	6	0 (0)	0	1	1 (1)	1
12	6	0 (0)	0	…	…	…
13	5	0 (0)	0	…	…	…
14	2	0 (0)	0	…	…	…
15	7	0 (0)	0	…	…	…
16	3	0 (0)	0	…	…	…
21	1	0 (0)	0	…	…	…
**Total**	256	19 (19)	27	77	24 (21)	32
**Infection rate (%)**	10.55			41.56		
**Potentially infective / Infected (%)**	100.00			87.50		

… not applicable (absence of live females);

^a^ two females anesthetized for dissection;

^b^ four females anesthetized for dissection;

^c^ presence of blood in the gut of one female;

pot. inf.: potentially infective; PCR: polymerase chain reaction.

In the experiments conducted with dogs, the infection rate was 10.55% (95% CI: 7.35% to 14.91%). Considering only the direct microscopic examination, the infection rate was 7.42% (95% CI: 4.65% to 11.53%), and all sand fly females developed late-stage infections with colonization at the stomodeal valve and anterior thoracic midgut (S1 Video). Moderate late-stage infections were observed in 89.47% of infected females (17/19; 95% CI: 82.43% to 96.51%). No heavy late-stage infections were observed. The sand fly females were experimentally infected in just one of the six replicates. On this occasion, the infection rate was 35.53% (27/76; 95% CI: 25.12% to 47.41%). A peritrophic matrix surrounding the bloodmeal was observed in the midgut of some females up to 5 days after feeding.

In the three experiments with hamsters infected with *L*. *amazonensis*, the infection rate was 41.56% (32/77; 95% CI: 31.21% to 52.71%). Experimental infection of the sand flies was successful in all three replicates. Among the 24 females with flagellate forms, three did not exhibit infective forms. The colonization of the stomodeal valve and/or the anterior thoracic midgut by metacyclic promastigotes was observed in 80.95% (17/21; 95% CI: 72.38% to 89.52%). Metacyclic forms in the foregut were observed in 19.05% (4/21; CI: 10.48% to 27.62%). Most infections were moderate (79.16%) in the infected flies. Female sand flies were found with their bloodmeal surrounded by a peritrophic matrix up to the fourth day. While one female sand fly exhibited blood in the gut, metacyclic forms were found in the head and stomodeal valve on day 3 after feeding (S2 Video). Furthermore, two female sand flies had heavy infection involving colonization of the stomodeal valve and the presence metacyclic promastigotes throughout the gut, from the foregut (head) to the distal portion of the abdominal midgut on day 5 after experimental infection (S3 Video).

**Fig 1:**
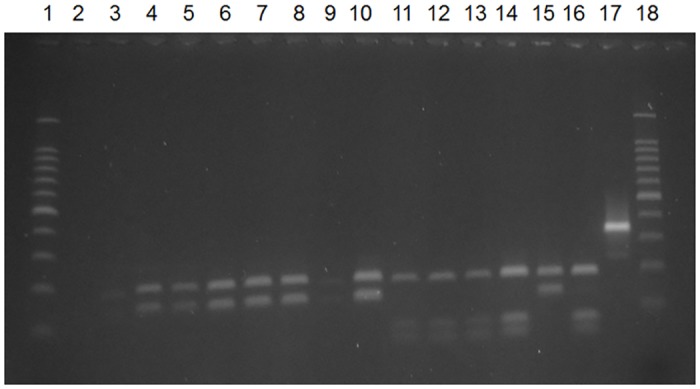
Digestion of amplified products of the ITS1 region of *Leishmania* with the *Hae*III restriction enzyme. 1: 100-bp ladder marker; 2: negative control; 3–10: sand flies fed on hamsters infected with *L*. *amazonensis*; 11–14: sand flies fed on dogs infected with *L*. *infantum*; 15: positive control *L*. *amazonensis* (IFLA/BR/1967/PH8); 16: positive control *L*. *infantum* (MHOM/BR/1972/BH46); 17: sample not digested by *Hae*III; 18: 100-bp ladder marker.

*Leishmania* DNA was detected by PCR in all female sand flies that were positive for flagellates during the direct microscopic examination. Moreover, 16 specimens that were considered negative during the direct exam were found to be positive in the PCR. The RFLP analysis performed by the digestion of the positive samples with the *Hae*III enzyme confirmed the species of *Leishmania* used for experimental infection, distinguishing *L*. *infantum* from *L*. *amazonensis* (Fig 1).

Metacyclic forms were observed in the stomodeal valve and anterior thoracic midgut on day 3 after xenodiagnoses for both species of *Leishmania*. Thus, the extrinsic incubation period in the sand fly was 3 days. Flagellate forms were found in females that had fed on dogs and hamsters up to the tenth and eleventh days, respectively.

### Survival

**Fig 2:**
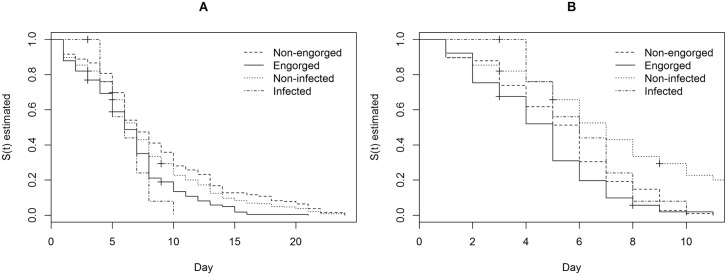
Survival estimates using the Kaplan-Meier estimator for engorged, non-engorged, infected, and non-infected females from cohorts exposed to dogs (A) and hamsters (B).

The survival functions for the groups analyzed were estimated using the nonparametric Kaplan-Meier estimator (Fig 2). The mean and median descriptive measures (Table 4) were obtained from the estimated curves. Both measures indicated that survival was lower in the group of engorged females, regardless of the source of blood and/or *Leishmania* infection. The log-rank test rejected the hypothesis of equality in the survival functions of the groups of engorged and non-engorged female sand flies, with *p*-values < 0.001 and equal to 0.0247 for the cohorts exposed to dogs and hamsters, respectively.

**Table 4:** Descriptive measures (in days) obtained using the Kaplan-Meier estimator, by host and species of *Leishmania*.

Measure	Dogs infected with *L*. *infantum*				Hamsters infected with *L*. *amazonensis*			
	Engorged		Infected		Engorged		Infected	
	Yes	No	Yes	No	Yes	No	Yes	No
Median	6.43	7.47	6.43	6.49	4.10	5.06	4.68	2.90
Mean	6.49	8.59	5.36	6.91	4.55	5.32	5.43	3.89

With respect to infected and non-infected female sand flies, the presence of the parasite did not appear to influence the daily probability of survival for those infected with *L*. *infantum*. The log-rank test (*p* = 0.278) did not reject the hypothesis of equality in the survival functions of these groups. However, female sand flies infected with *L*. *amazonensis* had a significantly greater probability of survival in comparison to those that had fed, but were not infected (*p* = 0.021).

Survival expectancy, given by the median time in which the infected females completed the gonotrophic cycle (or the expectation of a sand fly’s infective life), was 1.32 and 0.43 for *L*. *infantum* and *L*. *amazonensis*, respectively.

Cox regression models were used to study the relationship between the covariates on the survival rates. For the female sand fly group that was engorged after xenodiagnosis with dogs (Table 5), the likelihood ratio test showed that the inclusion of egg-laying time or the gonotrophic cycle (Model 4) made the estimated model significant (*p* = 0.001), i.e., at least one of the explanatory variables was statistically significant.

**Table 5:** Estimates obtained from the Cox regression models for the female sand fly group that was engorged after xenodiagnosis with dogs.

Model	Covariate	Estimate	Partial log-likelihood
1			
	None	-	-2956.112
2			
	*L*. *infantum* infection	0.427	-2954.219
3			
	*L*. *infantum* infection	0.371	-2953.591
	Egg-laying	0.246	
4			
	*L*. *infantum* infection	0.294	-2948.791
	Egg-laying	2.096	
	Egg-laying time	-0.250	
5			
	*L*. *infantum* infection	0.295	-2948.777
	Egg-laying	2.084	
	Egg-laying time	-0.253	
	Number of eggs	0.003	

However, as shown in Table 6, *L*. *infantum* infection was non-significant (*p* = 0.316). The final model is therefore shown in Table 7 without this variable. Thus, according to the results shown in this table, egg-laying contributes positively (beta value significant and greater than zero) to the lifetime of female sand flies infected by feeding on infected dogs, while egg-laying time reduces survival (beta value significant and less than zero). Based on the model, the other variables analyzed were not significant and did not play an important role in the survival of female sand flies infected by *L*. *infantum*.

**Table 6:** Estimates obtained for the Cox regression model adjusted by considering the covariates *L*. *infantum* infection, egg-laying, and egg-laying time.

Covariate	Estimate	Standard error	z	*p*-value
*L*. *infantum* infection	0.294	0.217	1.003	0.176
Egg-laying	2.096	0.627	3.466	<0.001
Egg-laying time	-0.250	0.089	-2.870	0.005

**Table 7:** Estimates obtained for the Cox regression model adjusted by considering the covariates egg-laying and egg-laying time.

Covariate	Estimate	Standard error	z	*p*-value
Egg-laying	2.233	0.613	3.643	<0.001
Egg-laying time	-0.259	0.088	-2.938	0.003

Cox regression models were also used to study the female sand fly group that was engorged after xenodiagnosis with hamsters (Table 8). In this case, no significant differences were found between the partial log-likelihood values. When assessing the largest difference between values (Model 4 in comparison to Model 3) using the likelihood ratio test, there was no significant *p*-value. Thus, the analyzed covariates did not play an important role in the survival of female sand flies infected by *L*. *amazonensis*.

**Table 8:** Estimates obtained from the Cox regression models for the female sand fly group that was engorged after xenodiagnosis with hamsters.

Model	Covariate	Estimate	Partial log-likelihood
1			
	None	-	-829.7007
2			
	*L*. *amazonensis* infection	-0.109	-829.570
3			
	*L*. *amazonensis* infection	-0.099	-829.517
	Egg-laying	-0.088	
4			
	*L*. *amazonensis* infection	-0.159	-827.710
	Egg-laying	2.234	
	Egg-laying time	-0.372	
5			
	*L*. *amazonensis* infection	-0.159	-827.708
	Egg-laying	2.203	
	Egg-laying time	-0.371	
	Number of eggs	0.005	

### Human and canine attractiveness to sand flies

Attractiveness of the host to the sand flies was evaluated by three different methods (Table 9). Using the method proposed by Pinto et al. [33], 48 replicates were performed (24 for each host) to evaluate human and canine attractiveness to *Lu*. *cruzi*. However, only five replicates had positive results for *Lu*. *cruzi*, as well as other species of sand fly, such as *Lutzomyia forattinii*, *Evandromyia corumbaensis*, *Evandromyia sallesi*, and *Micropygomyia peresi*. No sand flies were caught in the other 43 trials. A total of 81.56% of the specimens were collected in two replicates performed simultaneously: 595 (578 males and 17 females) at the tent with the human, and 604 (574 males and 30 females) at the tent with the dog.

**Table 9:** Attractiveness of hosts to sand flies, according to the different methods employed.

Method	N	*Lu*. *cruzi*		*Lu*. *forattinii*		*Ev*. *corumbaensis*		*Ev*. *sallesi*		*Mi*. *peresi*		Total
		M	F	M	F	M	F	M	F	M	F	
Tent (human)	24	588	23	…	01	01	…	…	01	…	01	615
Tent (dog)	24	573	32	01	…	…	04	…	…	…	…	610
Aspiration (dog and kennel)	04	45	164	…	…	…	…	…	…	…	…	209
Disney trap (dog)	06	27	4	…	…	…	…	…	…	03	02	36
**Total**	-	1235	221	01	01	01	04	00	01	03	03	1470

… not observed; N: number of replicates; M: male; F: female; *Lu*.: *Lutzomyia*; *Ev*.: *Evandromyia*; *Mi*.: *Micropygomyia*.

During the collection performed by aspiration on dogs, 209 specimens of *Lu*. *cruzi* (45 males and 164 females) were caught. Considering only the female sand flies, 131 were caught in the first trial within 1.5 hours (19:00 to 20:30), 29 were caught in the second trial over the same time period as in the first trial, four were caught in the third trial over a 5-hour period (18:00 to 23:00), and none were caught in the fourth trial over a 3-hour period (18:00 to 21:00). The collection was performed at the same residence and on the same animal on the first two occasions.

The collections using metallic discs (Disney trap) were made on six occasions. No sand flies were collected on two occasions, whereas 36 (31 specimens of *Lu*. *cruzi* and five specimens of *Mi*. *peresi*) were caught on the other occasions (Table 9).

### Experimental transmission of *Leishmania* spp.

Nine naive hamsters were exposed to *Lu*. *cruzi* females that had fed on infected hosts: six with females from the xenodiagnoses in dogs, and three in hamsters. We were only able to demonstrate the experimental transmission of *L*. *amazonensis*.

#### Leishmania infantum

Six naive hamsters were exposed to bites (second bloodmeal) from a total of 144 *Lu*. *cruzi* females engorged on dogs infected with *L*. *infantum* (first bloodmeal). The feeding rate during the second blood feeding was 49.31% for female sand flies that fed on susceptible hamsters. Among the female sand flies that fed (second bloodmeal), 18.31% were infected by the parasite (Table 1). However, none of the hamsters became infected after the follow-up, testing negative according to all three detection methods (direct parasitological test, isolation in the culture medium, and PCR).

#### Leishmania amazonensis

Three naive hamsters were exposed to bites from a total of 29 *Lu*. *cruzi* females engorged on infected hamsters (first bloodmeal). Fourteen of these female sand flies engorged on susceptible hamsters (second bloodmeal). Therefore, the feeding rate during the second blood feeding was 48.28% for females that fed on hamsters infected with *L*. *amazonensis*. Among the female sand flies that fed, 57.14% were infected by the parasite (Table 1).

The vector competence of *Lu*. *cruzi* through the experimental transmission of *L*. *amazonensis* was demonstrated in only one hamster, which became infected after exposure to the second bloodmeal of 21 females, six of which were engorged. This animal died 22 days after the second bloodmeal. It was the only animal to die before the established six-month follow up period. Thus, it was not possible to seed the tissue fragments (spleen) from this animal into the culture medium for the isolation of the parasite. However, amastigotes were found during the direct parasitological test (imprint of the spleen, S3 Fig), and *Leishmania* DNA was detected using PCR. The PCR-RFLP and the sequencing confirmed infection by *L*. *amazonensis*. During the physical exam, the animal did not exhibit specific symptoms attributable to infection by *Leishmania*. The remaining animals tested negative according to all three methods (direct parasitological test, isolation in the culture medium, and PCR).

## Discussion

The present study was conducted to investigate the ecological components of the parasite–vector interactions of *Lu*. *cruzi* with *L*. *infantum* and *L*. *amazonensis*. Initially, the project focused on *L*. *infantum*–*Lu*. *cruzi*. However, the presence of *L*. *amazonensis* DNA was detected in wild-caught females of *Lu*. *cruzi* during project execution [28]. We therefore decided to include this parasite in the study. Few studies have addressed issues inherent to the biology and behavior of *Lu*. *cruzi*, including its vectorial capacity and competence. Consequently, reports on parasite–vector interaction are not available, but are necessary to confirm a vector. The present results demonstrate that *Lu*. *cruzi* developed late-stage infections of both *L*. *infantum* and *L*. *amazonensis*, with colonization of the stomodeal valve. Thus, this study is the first to describe the parameters of the *Leishmania*–sand fly interaction involving *Lu*. *cruzi*, and the first to examine the experimental transmission of *Leishmania* by bites from this fly.

The proportion of engorged females ranged from 0.33 to 1.00, regardless of host (dog or hamster). Despite an equal total feeding rate in the tests performed with dogs and hamsters, greater variability in individual rates was found in the experiments involving dogs, with the feeding rate being inversely proportional to the number of sand flies released into the cage for the xenodiagnosis. Although environmental factors (such as temperature, humidity, and exposure time) influence the feeding rates of these insects, this finding might be explained by competition for space to land and feed on a host due to the higher density of insects in the cage [15].

The influence of temperature must be carefully evaluated in studies involving experimental infection, particularly as insects are poikilothermic, and their internal temperature varies considerably depending on the surrounding air temperature. This phenomenon might affect the parasite–vector interaction and the likelihood of exposure to infection based on food preference and biting rate on the host [37]. However, the role of the climate in parasite development in vector insects has received little attention [37]. In the present study, temperature and relative humidity ranged from 24.00 to 34.50°C and 29.00 to 100.00%, respectively, during the experiments with dogs. These parameters did not appear to significantly influence feeding rates (S1 Table).

Some authors have reported that the parasite might mold and manipulate the time of the bloodmeal of the sand fly vectors so that feeding coincides with the peak occurrence of infective forms plugging the anterior portion of the midgut as a result of the promastigote secretory gel, which might lead to multiple feeding attempts [38]. The estimated time for further bloodmeals is calculated by the duration of the gonotrophic cycle. In the present study, the median gonotrophic cycle was 6 days (range: 3 to 15 days) when the infective forms were already located in the anterior midgut and foregut. This finding is similar to that described by Galvis-Ovallos [16] for *Lu*. *longipalpis*, a species for which the median gonotrophic cycle was 5 days (range: 3 to 10 days).

Although membrane feeding is useful in studies of *Leishmania*–sand fly interaction and vector competence, feeding on infected animals is closer to natural conditions [20]. All dogs used in the present study for xenodiagnosis were symptomatic, and had positive direct parasitological findings for *Leishmania*. However, experimental infection of female sand flies was only detected on one occasion. Certain factors, separately or in combination, might explain the low infection rate, including infection time, cutaneous parasite load, skin condition of the mammalian host, and its immunological status [39].

This study used hamsters experimentally infected with *L*. *amazonensis* for xenodiagnosis to assess the infection of *Lu*. *cruzi*. It is worth noting that the localization of the lesions and the severity of infection in experimental hamsters might differ from those of native rodents naturally infected by *L*. *amazonensis*. Furthermore, access of sand flies to active lesions on anaesthetized hamsters might also be different from what occurs under natural conditions. However, the parasite–sand fly interaction after xenodiagnosis could be used to infer the actual status under natural conditions.

Our results demonstrate an infection rate of 10.55% for *L*. *infantum* and 41.56% for *L*. *amazonensis*. Montoya-Lerma et al. [40] employed four methods to compare the vector competence of *Lu*. *longipalpis* and *Pintomyia evansi* for *L*. *infantum*. When exposing only the ear or lower abdominal region of polysymptomatic and oligosymptomatic dogs to canine visceral leishmaniasis, the mean infection rates ranged from zero to 6.80% for *Lu*. *longipalpis* and zero to 4.40% for *Pi*. *evansi* over a 20-min exposure time. *Lu*. *longipalpis* fed more avidly than *Pi*. *evansi*, and exhibited heavy infection. In contrast, *Pi*. *evansi* only developed mild infection. In their experiments with hamsters, the infection rates were significantly lower for both species of sand fly, contradicting the present findings.

Diniz et al. [15] obtained infection rates ranging from 6.20 to 34.40% when investigating only positive female sand flies during the dissection of cohorts from six species of sand fly exposed to hamsters infected with *L*. *braziliensis*. For *Lu*. *longipalpis*, the infection rates based on the dissection and microscopic examination of female sand flies exposed to dogs with canine VL ranged from zero [41] to 42.00% [42]. The infection rates in the present study were calculated based on positive PCR results. It is possible that the 14 samples that tested positive in the PCR, but negative during the direct microscopic exam, died during the night and were dissected some hours after death when the parasites were already dead. Although the parasite–vector–host combinations differ, they might explain the differences in infection rates among studies. When considering only dissection and direct microscopic examination, the infection rate in the present study was 7.42 and 31.17% for cohorts that fed on dogs naturally infected with *L*. *infantum* and hamsters experimentally infected with *L*. *amazonensis*, respectively.

Results of experimental sand fly infection should be interpreted with caution, as infection is influenced by a number of factors such as the dose of the parasite [40,43] and the rate of parasitic reproduction. Even in cases of co-evolutionary associations, variable degrees of refractivity or permissiveness are expected [40]. The higher infection rate found for the combination of *Lu*. *cruzi* and *L*. *amazonensis* might be explained by the degree of cutaneous parasitism in hamsters compared to dogs infected with *L*. *infantum*. Although it was not possible to quantify cutaneous parasitism in the hosts, the nodular lesions on the hamsters might have contained higher concentrations of amastigotes, thus increasing the chances of infection by female sand flies. Stamper et al. [43] have pointed out that in nature, sand flies likely become infected with varying doses of parasites depending upon their feeding behavior and the concentration of parasites in the lesion or blood upon which they feed. When studying the relevance of infectious bite sites in the transmission of *L*. *infantum* back to vector populations, Aslan et al. [44] highlighted the fact that transmissibility from infected dogs to sand flies remains poorly understood, and it is complicated by the unknown location and frequency of infected bites in field settings.

Taking into account that *Lu*. *cruzi* is phylogenetically very closely related to *Lu*. *longipalpis* and the two species constitute a species complex [22,45,46], the laboratory permissiveness exhibited by *Lu*. *longipalpis* may be associated with that of *Lu*. *cruzi*. Perhaps it is possible that both species in this group are permissive vectors.

The developed and established late-stage infections of *L*. *amazonensis* in *Lu*. *cruzi* suggest that the mechanism of adhesion of the protozoan to the intestinal epithelium of sand flies is not species-specific. Consequently, it is independent of lipophosphoglycan (LPG) on the surface of *Leishmania* [47,48], as occurs in other vectors considered permissive [19,49]. Intraspecific variation in the LPG of *L*. *infantum* [50] and *L*. *amazonensis* [51] did not affect their development in the vectors *L*. *longipalpis* and *Migonemia migonei*.

Extrinsic incubation time varies depending on the species of *Leishmania* and sand fly, as well as on environmental conditions [19,48]. For combinations of *Lu*. *longipalpis* with *L*. *infantum* [52] and *L*. *mexicana* [19], metacyclic promastigotes occur in the anterior midgut from day 4 after the bloodmeal on an experimentally infected source. Rogers et al. [19] found low quantities of metacyclic promastigotes in the middle portion of the midgut from day 3 after feeding. However, due to the location, these forms might not play a significant role in transmission. For combinations of six species of sand fly infected with *L*. *braziliensis*, the extrinsic incubation period ranged from 4 to 6 days [15]. Our data showed that metacyclic forms were found in the anterior thoracic midgut on day 3 after xenodiagnoses for both *Leishmania*–sand fly combinations.

The effect of infection by *Leishmania* on the longevity and fecundity of sand flies has not yet been clarified [25]. Agrela and Feliciangeli [53] showed that the infection of *Lu*. *longipalpis* and *Lutzomyia pseudolongipalpis* by *L*. *infantum* and *L*. *braziliensis* reduced survival and life expectancy, and decreased fertility and fecundity (egg production). El Sawaf et al. [54] found that infection by *L*. *infantum* and *L*. *major* significantly reduced the longevity and fecundity of *Phlebotomus papatasi* and *Phlebotomus langeroni* in comparison with engorged, non-infected females. Although the mechanism by which survival and egg-laying decreased was not clear, the authors suggested that lesions in the stomodeal valve induced by the chitinase produced by the parasite might be a contributing factor [55,56]. Some researchers also cite the stress of infection as a possible cause of reduced longevity [38]. In contrast, Sant’anna et al. [57] found that the infection of *Lu*. *longipalpis* by *L*. *mexicana* conferred protection against bacterial infection, which could indirectly influence insect survival. The present data demonstrated a significant difference in the survival function between engorged and non-engorged females, suggesting that the bloodmeal itself reduced the probability of survival. This finding might be explained by egg-laying, as only engorged females are able to mature and lay eggs, with this process normally causing stress and exhaustion that culminates in death [58]. This hypothesis is supported by the marked drops in both survival curves for engorged females on day 5 after the bloodmeal (Fig 2A and 2B), which was 1 day before the end of the median gonotrophic cycle. Infection by *L*. *infantum* did not influence the survival of engorged females. In contrast, females infected by *L*. *amazonensis* had a significantly greater probability of survival than engorged, non-infected females. Further studies with a larger sample size are needed to confirm these findings.

Regardless of *Leishmania* infection, the length of the sand fly life cycle varied according to the blood source. Females that fed on dogs showed greater longevity than those that fed on hamsters. This variation in behavior related to the blood source was observed for another sand fly species, *Mg*. *migonei* [59]. These data might be useful in determining the best blood source for maintaining an insect colony.

Concerning canine and human attractiveness to sand flies, the three methods produced different results in relation to the quantity and frequency of the specimens collected. The experiments conducted with tents led to the greatest yield and diversity of species. However, positive results were only obtained in five of the 48 replicates. The 1,199 specimens captured in two simultaneous replicates might have been the result of a population explosion on that day, as all females captured were nulliparae, and were not engorged. These results raise the question of whether this methodological approach is adequate for the study of attractiveness. Pinto et al. [33] compared the attractiveness of human odors and carbon dioxide (CO_2_) for *Nyssomyia intermedia* and *Nyssomyia whitmani* using the same tent method. For both species, the proportion of human attractiveness attributable to CO_2_ was significantly lower for males. The attractiveness of other hosts, such as chickens and dogs, to sand flies is frequently described in the literature [11]. When the attractiveness of a given vertebrate species to a blood-sucking insect is investigated, that is an indirect evaluation of the biting rate on the host, which is one of the parameters used to estimate vectorial capacity. This parameter is defined as the daily number of potentially infective bites that a population of vectors might make when feeding on a source of infection [13,14].

Our study demonstrated the vector competence of *Lu*. *cruzi* for *L*. *amazonensis* experimentally. Even though the experimental transmission of *L*. *infantum* was not successful, the vector competence of *Lu*. *cruzi* was naturally demonstrated for *L*. *infantum* by Oliveira et al. [35]. Besides being labor intensive, the experimental transmission of *Leishmania* presents difficulties, such as the high mortality rate of females following egg-laying, which impedes the second bloodmeal and the gonotrophic concordance of many species that only require one bloodmeal for the maturation of the ovaries and egg-laying. The low proportion of infected female sand flies that take the second bloodmeal must also be considered [18]. In the present study, mean feeding rates during the second bloodmeal ranged from zero to 100%. Although not evaluated systematically, many female sand flies did not become completely engorged during the second bloodmeal, as previously observed for *Lu*. *longipalpis* females infected by *L*. *amazonensis* [18]. Stamper et al. [43] explored the influence of sand fly oviposition status on transmission rate. They showed that flies that had oviposited were more likely to take a bloodmeal than flies with retained eggs, and this suggests that flies feed more efficiently or pursue a second bloodmeal more aggressively after the egg-laying. This observation may partially explain the failure of experimental transmission of *L*. *infantum*, since the challenge was performed on day 4 after the xenodiagnosis and some females might not have completed the gonotrophic cycle.

It has been shown experimentally that the number of parasites and the proportion of midgut promastigotes that have differentiated into metacyclic forms in the infectious feed affects transmission efficiency [43]. It is possible that the greater infection rate of females fed on *L*. *amazonensis*–infected hamsters is linked to the greater number of parasites observed in infected flies and the successful experimental transmission.

Oliveira et al. [35] reported the occurrence of a hamster that was naturally infected by *L*. *amazonensis* in Corumbá. The naturally infected animal exhibited no clinical signs of infection by *L*. *amazonensis*, such as the nodular lesions, corroborating to our laboratorial findings. Furthermore, the DNA of the parasite was identified based on spleen fragments, suggesting possible visceralization. Oliveira et al. [35] supported the hypothesis proposed by Sherlock [41] and Warburg et al. [60]. Specifically, elements of the saliva of *Lu*. *longipalpis* might change the behavior of *L*. *amazonensis*, altering its tropism so that the parasite causes visceral, rather than cutaneous, leishmaniasis. Considering that *Lu*. *cruzi* is regarded as a sibling species within the *Lu*. *longipalpis* complex [22,45,46], it is possible that *Lu*. *cruzi* shares the same behavior.

During the study period, our research group has also studied the monthly distribution and the seasonal dynamics of sand flies in Corumbá. *Lu*. *cruzi* was the most frequently collected species, accounting for 93.94% of all sand flies, and it was found in all collection months, including the dry and rainy seasons. These observations indicate a continuous risk of infection throughout the entire year [61].

It is important to study the ecology and evaluate the parameters involved in the vectorial capacity of insects. Such variables can be used (either alone or in conjunction) as predictors during the creation of mathematical models of parasite transmission scenarios [13]. Here, based in laboratory and field data, we evaluated all the parameters linked to the vectorial capacity of an insect, as proposed by Garret-Jones [12] and Macdonald [14].

Regarding the vector incrimination of *Lu*. *cruzi* to *L*. *infantum*, evaluated through the vectorial capacity parameters, in this study three of the six Killick-Kendrick criteria to incriminate a vector [10] were met. For the first criterion, *Lu*. *cruzi* is attracted to humans and dogs; for the third, wild *Lu*. *cruzi* were collected in an endemic area for human and canine VL; and for the fifth, the parasite developed late-stage infection and colonized the stomodeal valve of the fly. The second and forth criteria are related to the repeated isolation and identification of the same species of *Leishmania* from female sand flies as is found in the VL cases, and the density and rate of natural infection by *Leishmania*. These criteria were met by Santos et al. [4] and Pita-Pereira et al. [9]. The sixth criterion has not been met experimentally here, but previously our study group demonstrated the natural transmission *L*. *infantum* by bite of wild-caught *Lu*. *cruzi* to a naive host. Considering the Killick-Kendrick criteria and based on the presented evidence, *Lu*. *cruzi* can be incriminated as a vector of *L*. *infantum*.

For *L*. *amazonensis*, only a few vector capacity parameters could be evaluated, because its possible wild reservoir in the study area is unknown. However, we have been able to demonstrate important biological aspects that can be used for vector incrimination, such as the experimental transmission of the parasite.

In conclusion, the experimental infection of *Lu*. *cruzi* in this study provided novel insights into the components involved in its vectorial capacity for *Leishmania*. We present strong evidence that supports *Lu*. *cruzi* as a vector of *L*. *infantum*. The permissive nature of *Lu*. *cruzi* was suggested by the development of late-stage infections of *L*. *amazonensis*, and its experimental transmission to a naïve host. The present data indicate the need for further studies to evaluate the transmission dynamics of the parasites in regions with records of *Lu*. *cruzi*, as well as the identification of the species of *Leishmania* in possible reservoirs, other than dogs, in endemic areas and locations of sporadic transmission.

## Supporting information

S1 Fig Nylon cage used for xenodiagnosis with dogs. (A) Schematic; (B) Side view; (C) 3-dimensional view. Points 1–2 and 3–4 are linked by a meter-long double-sided zipper to allow opening and closing from both inside and outside.
(PDF)

S2 Fig Tent used for experiments on human and canine attractiveness to sand flies. (A) Front view– 1) device for the exiting of air (screened 1-m PVC tube); 2) device for the entry of air (screened 1-m PVC tube) with cooler (not shown in figure) to adjust velocity; (B) Schematic of tent.
(PDF)

S3 Fig Imprint of the spleen of the hamster showing amastigotes of *L*. *amazonensis*. (PDF)

S1 Table Temperature, relative humidity, and feeding rate during xenodiagnosis, by infected host. (PDF)

S1 Video *Lu*. *cruzi* infected by *L*. *infantum* on day 3 after xenodiagnosis. (MP4)

S2 Video *Lu*. *cruzi* infected by *L*. *amazonensis* on day 3 after xenodiagnosis. (MP4)

S3 Video Heavy infection of *Lu*. *cruzi* infected by *L*. *amazonensis* on day 5 after xenodiagnosis. (MP4)
