# Supplementary material for: Experimental infection and transmission of Leishmania by Lutzomyia cruzi (Diptera: Psychodidae): Aspects of the ecology of parasite-vector interactions
Source: PLoS Negl Trop Dis. 2017 Feb 24;11(2):e0005401. doi: 10.1371/journal.pntd.0005401 (PMC5342273; doi:10.1371/journal.pntd.0005401)

Supplementary Fig. S1. Nylon cage used for xenodiagnosis with dogs.

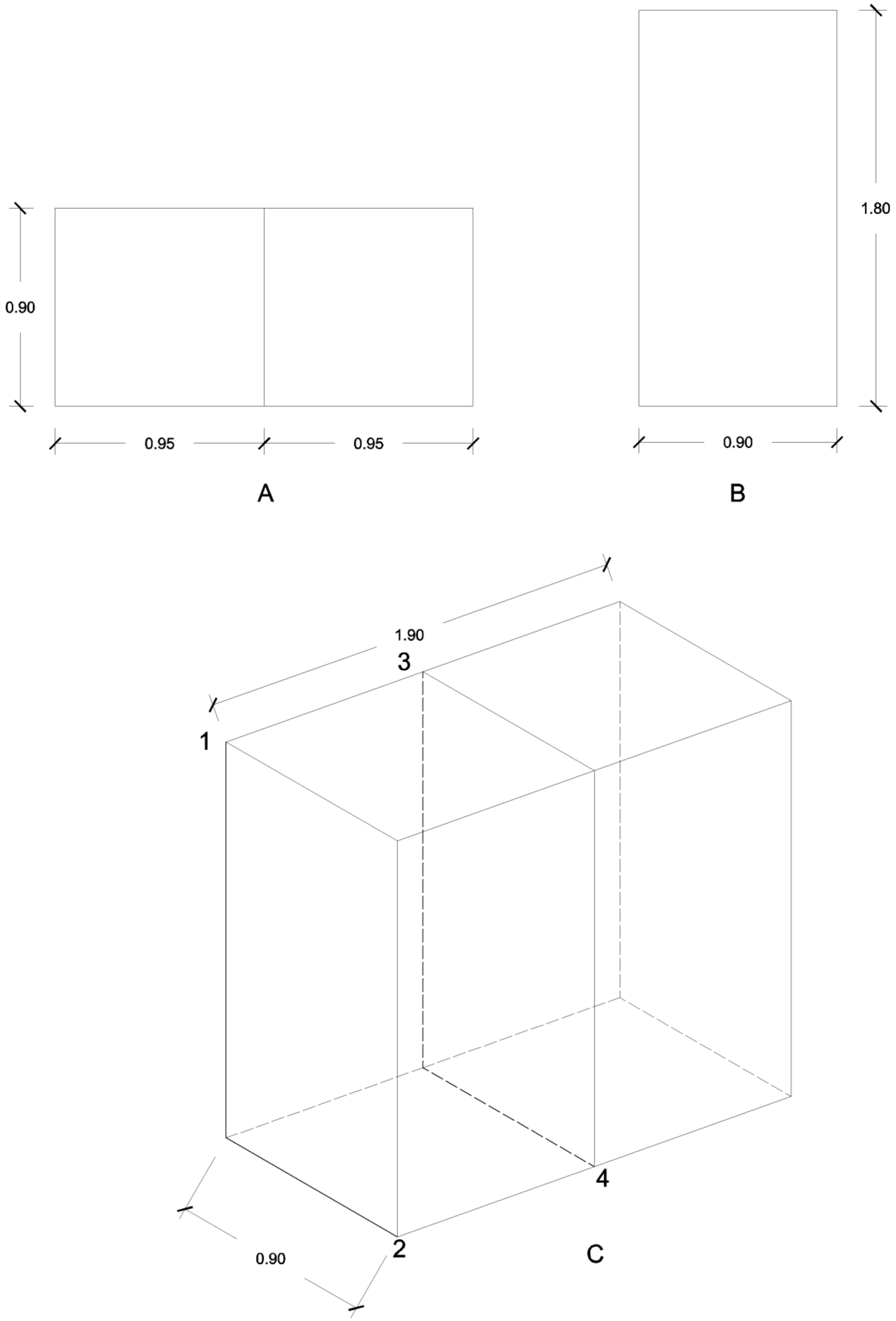

(A) Schematic; (B) Side view; (C) 3-dimensional view – points 1-2 and 3-4 linked by meter-long double-sided zipper to allow opening and closing from inside and outside.

Real Images:

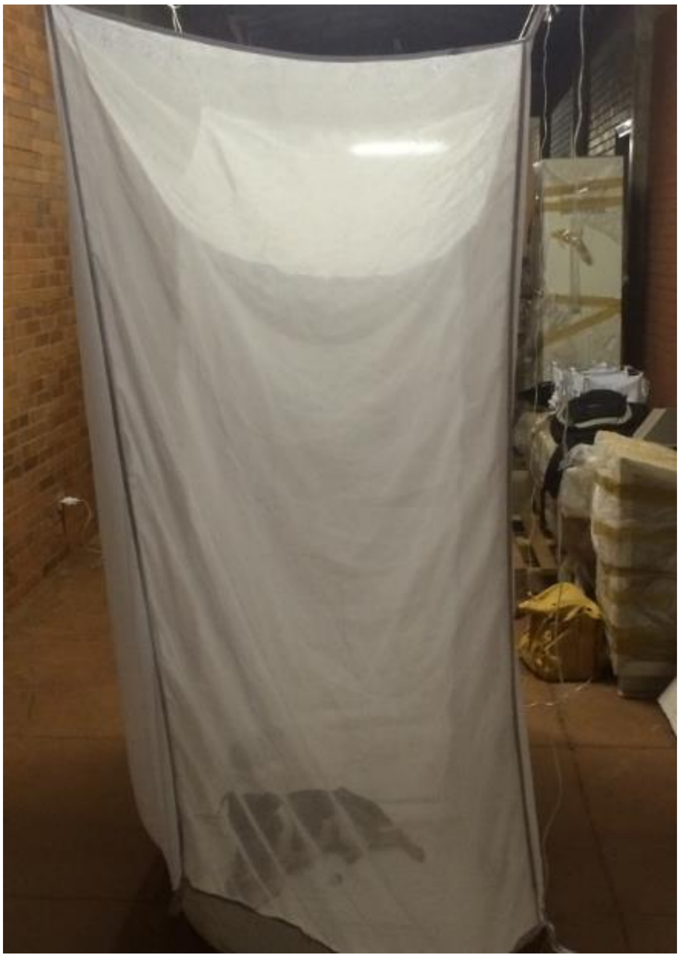

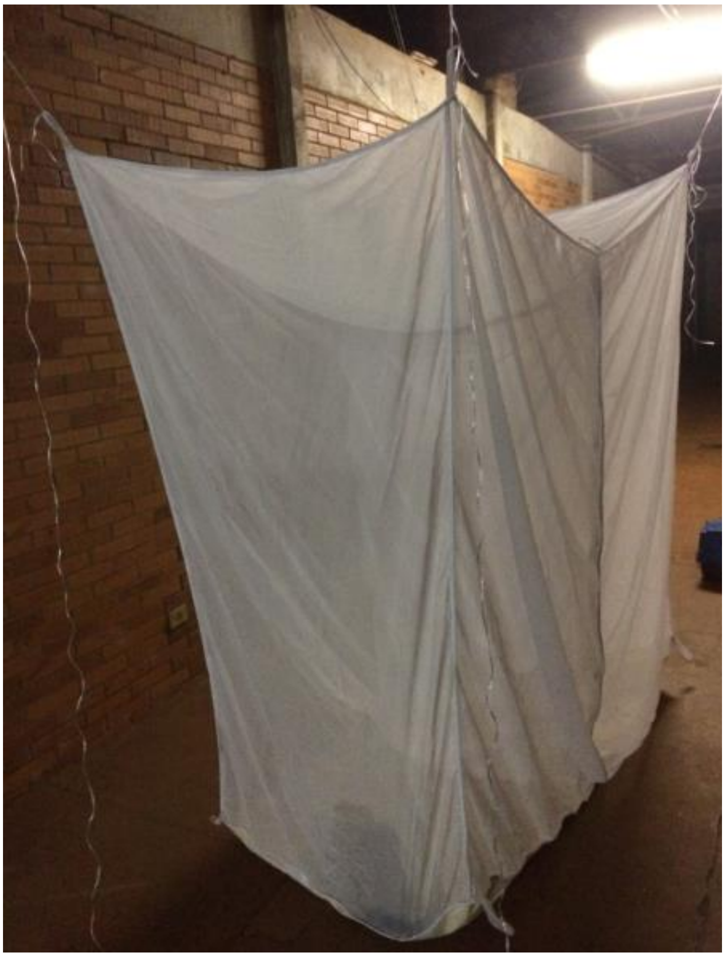

Supplement: S1 Fig — (A) Schematic; (B) Side view; (C) 3-dimensional view. Points 1–2 and 3–4 are linked by a meter-long double-sided zipper to allow opening and closing from both inside and outside. (PDF) [file pntd.0005401.s001.pdf]
