# Supplementary material for: Experimental infection and transmission of Leishmania by Lutzomyia cruzi (Diptera: Psychodidae): Aspects of the ecology of parasite-vector interactions
Source: PLoS Negl Trop Dis. 2017 Feb 24;11(2):e0005401. doi: 10.1371/journal.pntd.0005401 (PMC5342273; doi:10.1371/journal.pntd.0005401)

Supplementary Fig. S2. Tent used for experiments on human and canine attractiveness to sand flies.

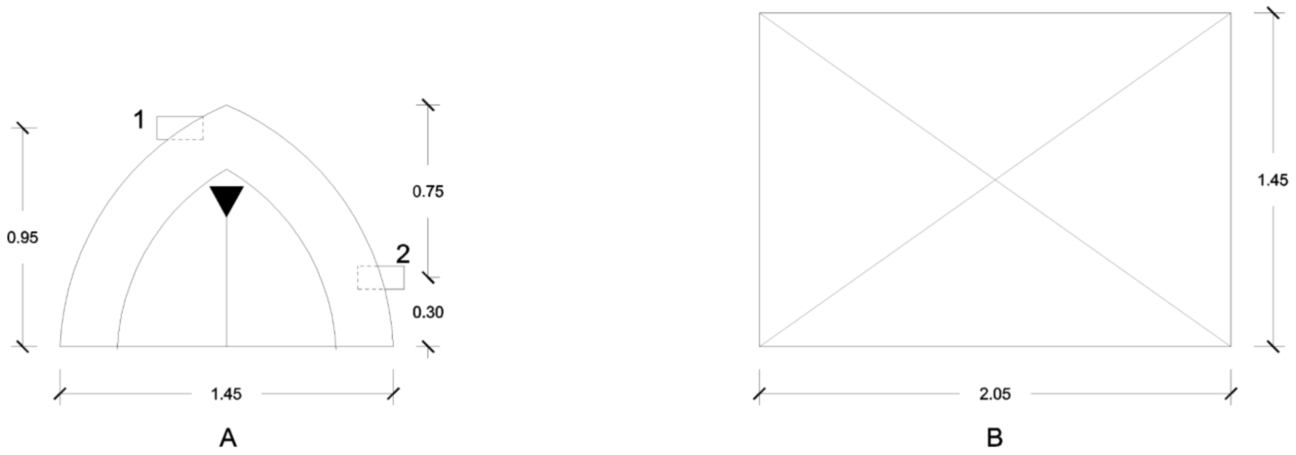

(A) Front view – 1) device for the exiting of air (screened 1-m PVC tube); 2) device for the entry of air (screened 1-m PVC tube) with cooler (not shown in figure) to adjust velocity; (B) Schematic of tent.

Real Images:

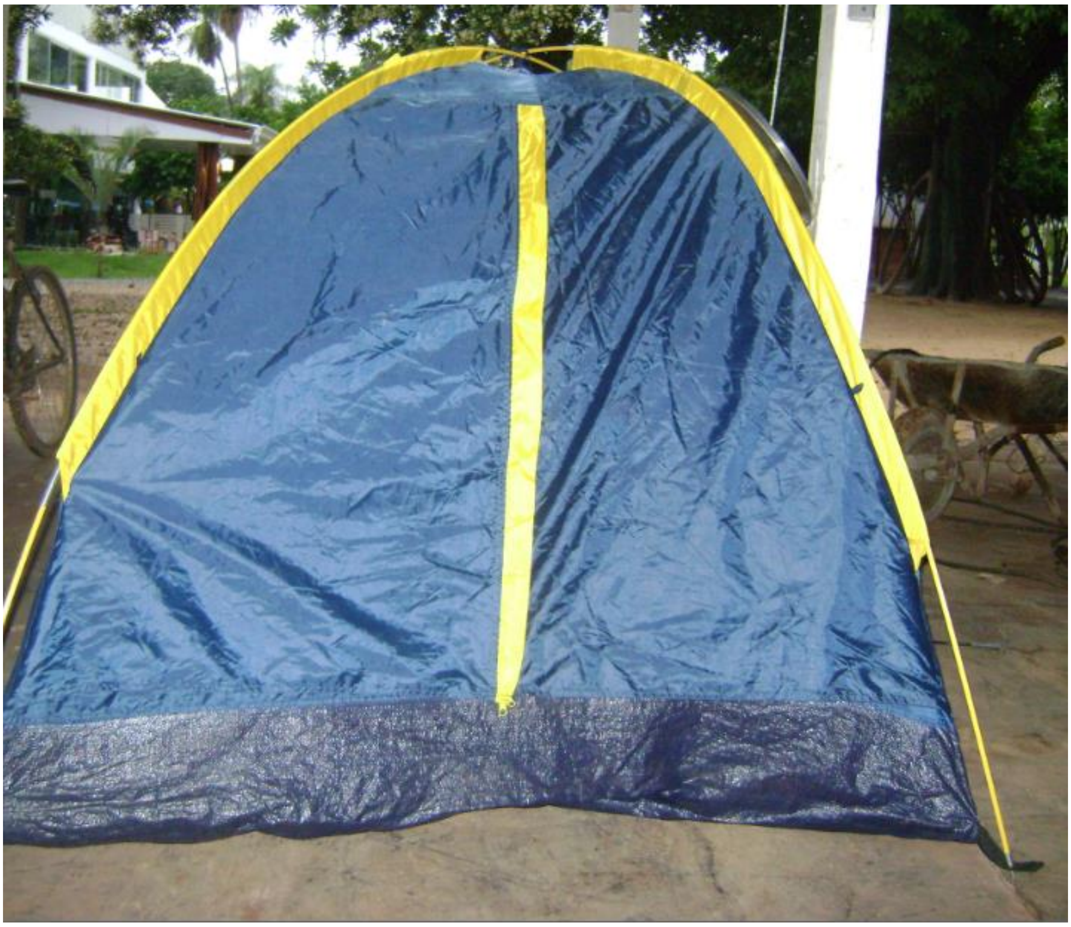

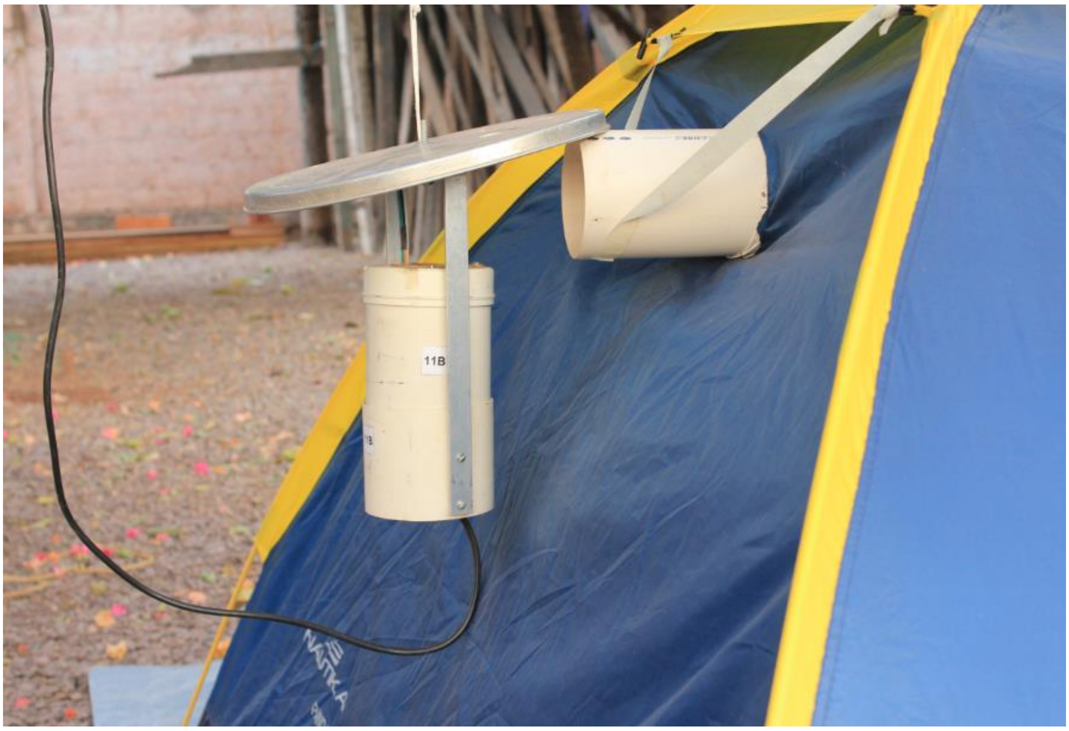

Supplement: S2 Fig — (A) Front view– 1) device for the exiting of air (screened 1-m PVC tube); 2) device for the entry of air (screened 1-m PVC tube) with cooler (not shown in figure) to adjust velocity; (B) Schematic of tent. (PDF) [file pntd.0005401.s002.pdf]
