# Supplementary figures and images for: Experimental infection and transmission of Leishmania by Lutzomyia cruzi (Diptera: Psychodidae): Aspects of the ecology of parasite-vector interactions
Source: PLoS Negl Trop Dis. 2017 Feb 24;11(2):e0005401. doi: 10.1371/journal.pntd.0005401 (PMC5342273; doi:10.1371/journal.pntd.0005401)

**Supplementary Fig. S3. Imprint of the spleen of the hamster showing amastigotes of *L. amazonensis*.**

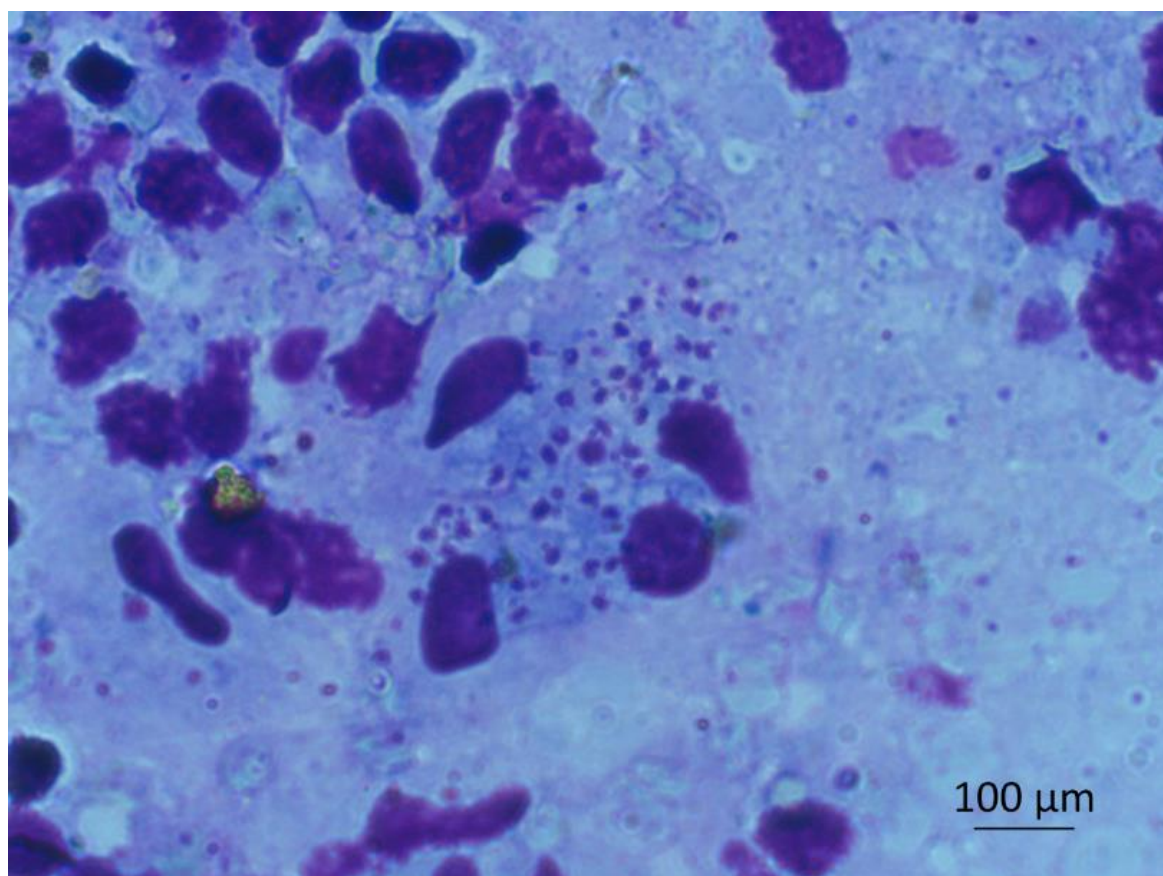

Supplement: S3 Fig — (PDF) [file pntd.0005401.s003.pdf]
