# Supplementary material for: Experimental infection and transmission of Leishmania by Lutzomyia cruzi (Diptera: Psychodidae): Aspects of the ecology of parasite-vector interactions
Source: PLoS Negl Trop Dis. 2017 Feb 24;11(2):e0005401. doi: 10.1371/journal.pntd.0005401 (PMC5342273; doi:10.1371/journal.pntd.0005401)
Supplement: S1 Table — (PDF) [file pntd.0005401.s004.pdf]

Supplementary Table S1. Temperature, relative humidity and feeding rate during xenodiagnosis according the infected hosts.

**Infected host**	**Date (AAAA/MM/DD)**	**Temperature (°C)**	**Relative humidity (%)**	**Feeding rate (%)**
Dog				
	2013/09/06	33.0	29.0	75.0
	2013/10/15	24.0	100.0	63.6
	2013/11/22	30.0	51.0	80.0
	2013/11/29	34.5	38.0	100.0
	2014/04/07	33.0	50.0	53.1
	2014/05/08	28.0	56.0	32.8
Hamster				
	2014/04/10	30.5	71.0	38.9
	2014/06/04	28.0	67.0	39.0
	2014/06/27	28.5	52.0	42.9

Note: all the experiments (replicates) were performed between 18:00 and 19:00.
